# Latent profiles and network analysis of emotional behaviors in preschool children after congenital heart disease surgery

**DOI:** 10.3389/fpubh.2026.1914221

**Published:** 2026-10-02

**Authors:** Qi Xu, Xiaotong Zhang, Yuxin Li, Jing Liu, Minmin Li

**Affiliations:** 1 Department of Neonatology, Shandong Provincial Hospital Affiliated to Shandong First Medical University, Jinan, China; 2 The School of Nursing, Shandong First Medical University, Jinan, China

**Keywords:** congenital heart disease, emotional and behavioral problems, family hardiness, latent profile analysis, network analysis, parenting stress, preschool children

## Abstract

**Background:**

Emotional and behavioral problems are prominent and highly heterogeneous among preschool children after congenital heart disease surgery, but their latent subgroup characteristics and core symptoms remain unclear. The associations between the family psychological environment (e.g., parenting stress, family hardiness) and children’s emotional and behavioral outcomes also need further clarification.

**Objective:**

To identify heterogeneous subgroups and central symptoms of emotional and behavioral problems in preschool children after congenital heart disease surgery, and to examine the associations between parenting stress, family hardiness, and children’s emotional and behavioral outcomes.

**Methods:**

A cross-sectional survey was conducted among 273 mothers of children aged 3–6 years after congenital heart disease surgery. Latent profile analysis was used to identify subgroups, and network analysis was applied to construct association networks of symptoms and related factors.

**Results:**

Two subgroups were identified: the well-adjusted subgroup (82.1%) and the maladjusted subgroup (17.9%). Hyperactivity/inattention was the node with the highest strength centrality in both subgroups. Parenting stress was independently associated with emotional and behavioral problems (OR = 1.086), while family hardiness was independently associated with a lower risk (OR = 0.907). The “challenge” dimension of family hardiness showed the highest centrality in the well-adjusted subgroup, whereas the “control” dimension showed the highest centrality in the maladjusted subgroup.

**Conclusion:**

Emotional and behavioral problems in preschool children after congenital heart disease surgery show significant heterogeneity. Different dimensions of family hardiness may serve as candidate targets for future intervention research, and early identification and management of hyperactivity/inattention should be prioritized.

## 1 Introduction

Congenital heart disease (CHD) is a congenital malformation caused by abnormal cardiovascular development during the fetal period, and is the most common type of birth defect in newborns (1). Approximately one in every 100 live births is affected (2). With continuous advances in medical technology, the survival rate of children with CHD has significantly improved (3), and the clinical focus has gradually shifted from disease treatment to long-term quality of life (4). However, multiple factors—such as the complexity of the disease itself, repeated hospitalizations, surgical trauma, and long-term rehabilitation management—pose unique physiological and psychological challenges for these children, making their risk of emotional and behavioral problems significantly higher than that of healthy peers (5). Previous studies have shown that up to 41% of children with CHD exhibit behavioral and/or emotional problems (6), characterized by withdrawal, emotional instability, and poor social adaptability, with an 8–12% increased risk of behavioral problems compared to healthy children (7). Moreover, higher disease severity and more frequent surgeries and hospitalizations are associated with more pronounced problems (8). These issues not only directly affect their quality of life during childhood but may also have lasting negative impacts on long-term social adaptation and mental health (9).

Emotional and behavioral problems (EBPs) encompass multiple dimensions, including emotional symptoms, conduct problems, hyperactivity/inattention, and peer relationship problems (10). These problems not only affect children’s immediate physical and mental health and social development but, if left unaddressed, may persist into adulthood, leading to chronic psychological issues such as anxiety and depression, and seriously impairing lifelong social adaptation and quality of life (11). The preschool period is a critical window for the development of emotional regulation, personality traits, and social adaptability in children (12). If emotional and behavioral problems at this stage are not effectively managed, they will significantly increase the likelihood of maladaptive behaviors during adolescence (9).

The occurrence of emotional and behavioral problems in children is influenced by multiple factors, including genetics, disease characteristics, and family environment (13). Family is a central source of support for children’s development. For children with CHD, however, the complexity of the disease itself, repeated medical experiences, and long-term rehabilitation needs may restrict their access to social and emotional support normally obtained from peers and schools, thus rendering the family an irreplaceable role in their psychosocial adaptation (14). When facing stress, families do not passively endure it; rather, they mobilize existing functional patterns, appraise stressors, integrate internal and external resources, and, through the interplay of problem-solving and coping strategies, move toward adaptation or maladaptation—when maladaptation occurs, new family crises emerge, manifested as emotional and behavioral problems in the child (15). The Resiliency Model of Family Stress, Adjustment, and Adaptation proposed by McCubbin (15) provides a systematic description of this dynamic process. According to the logic of this model, parenting stress is a key stressor faced by the family system, family hardiness is an important internal protective resource, and the child’s emotional and behavioral problems are essentially the outcome of family maladaptation.

Specifically, parenting stress refers to the difficulties and challenges perceived by mothers in the process of raising children. Research has found that high parenting stress reduces maternal parenting sensitivity, disrupts mother–child interaction patterns, and consequently increases the risk of emotional and behavioral problems in children (16). Family hardiness, as the core capacity of families to cope with adversity, refers to the family’s potential to maintain functional stability and recover from dysfunctional states under pressure and difficult circumstances (17). It can buffer the negative impact of stress by stabilizing the family atmosphere, promoting positive communication, and enhancing problem-solving efficacy (18), thereby providing a safe and supportive psychological development environment for the child. This aligns with the concept of family resilience proposed by Walsh (49). When stressors exceed the family’s capacity to regulate resources, the family falls into maladaptation, ultimately affecting the child’s emotional and behavioral outcomes.

However, existing studies have largely focused on the overall linear relationships among parenting stress, family hardiness, and emotional and behavioral problems in children, overlooking individual differences in the manifestation of these problems. Moreover, they have failed to deeply reveal the intrinsic mechanisms linking the family psychological environment and the dimensions of the child’s emotional and behavioral problems under different adaptation states, resulting in a lack of targeted clinical interventions and unsatisfactory outcomes. Latent profile analysis, as an emerging categorical statistical method, can identify latent homogeneous subgroups based on variable characteristics, providing an effective perspective for analyzing heterogeneous issues. Network analysis can visually present the strength of associations and core nodes among variables, offering a new research approach to uncover the internal structure and influence mechanisms of emotional and behavioral problems.

Therefore, this study focuses on preschool children after CHD surgery, and, based on the Resiliency Model of Family Stress, Adjustment, and Adaptation (19), aims to identify the heterogeneous characteristics and risk factors of emotional and behavioral problems in this population, to reveal the pathways through which the family psychological environment affects children’s emotional and behavioral outcomes, and to provide a scientific basis for developing stratified, individualized family psychosocial interventions. Ultimately, this study seeks to achieve early identification and precise intervention for emotional and behavioral problems in children with CHD, thereby promoting their physical and mental health and social adaptation abilities.

## 2 Methods

### 2.1 Participants and procedure

This study adopted a cross-sectional survey design. Participants were recruited from a tertiary hospital in Jinan between August 2025 and March 2026.

(1) Inclusion criteria for children: ① diagnosed with congenital heart disease and having undergone surgery; ② aged 3–6 years at the time of the survey; ③ free from major neurological disorders (e.g., cerebral palsy, active epilepsy, genetic neurodevelopmental syndromes such as chromosome 22q11.2 microdeletion syndrome and Down syndrome, and severe developmental delay) or other severe psychiatric diseases.

(2) Inclusion criteria for mothers: ① living with the child and primarily responsible for the child’s care; ② normal cognitive, reading, and communication abilities; ③ informed consent and voluntary participation in this study. Exclusion criteria: ① cognitive or language impairments; ② occurrence of major traumatic events in the past 6 months, such as severe natural disasters, sudden death of a family member, or unexpected accidents.

Regarding CHD diagnoses and surgical criteria: This study included all children who had undergone surgical treatment for congenital heart disease, encompassing both simple lesions (e.g., ventricular septal defect, atrial septal defect, patent ductus arteriosus) and complex lesions (e.g., tetralogy of Fallot, pulmonary atresia, double outlet right ventricle, transposition of the great arteries, hypoplastic left/right heart syndrome, total anomalous pulmonary venous return, complete atrioventricular septal defect, interrupted aortic arch). No upper limit was set for the number of surgeries, and enrolled children varied from one to multiple operations to reflect real-world clinical heterogeneity.

Clinical status at the time of assessment: At the time their mothers completed the questionnaires, all enrolled children had been discharged from the hospital and were in the postoperative recovery period.

Before the survey began, the purpose and procedures of this study were fully explained to the children’s mothers. Emphasis was placed on the voluntary nature of participation. Those who agreed to participate were asked to provide formal written informed consent. Data collection was conducted by trained investigators in the research team through personal telephone interviews or paper questionnaires. These investigators possessed extensive knowledge of congenital heart disease nursing and good research skills. The participants completed the entire survey independently. For those unable to complete the survey independently, the researcher read each question along with the corresponding response options, allowed the participant to answer according to their personal circumstances, and assisted in recording the responses. Any incomplete items were confirmed with the participant on site to ensure data completeness.

### 2.2 Sample size

Based on empirical guidelines (20), the sample size should be estimated as 5 to 10 times the number of independent variables in the questionnaire. There were 19 independent variables in this study. Considering a possible 10–15% rate of invalid questionnaires (21), at least 224 participants were required, and the final sample of 273 mothers met this requirement. In addition, according to the Pairwise Markov Random Field (PMRF), the parameters to be estimated for constructing the network model are the sum of threshold parameters and pairwise association parameters. In this study, we used one variable from the Parenting Stress Scale, three dimensions from the Family Hardiness Scale, and four dimensions from the Strengths and Difficulties Questionnaire (SDQ), resulting in 8 threshold parameters. The number of pairwise association parameters was [8 × (8–1)/2 = 28], giving a total of 36 parameters. Therefore, each subgroup in the network analysis required a minimum sample size of at least 36 participants.

### 2.3 Instruments

#### 2.3.1 The demographic and clinical measures

Self-reported demographic characteristics included child’s age, sex, type of medical insurance, number of hospitalizations for congenital heart disease, number of surgeries, receipt of financial support, place of residence, as well as mother’s age, educational level, occupation, presence of chronic illness, annual household income, number of children in the family, presence of illness in other children in the family, and family type. Medical information, including CHD classification, time of diagnosis, and number of coexisting CHD defects, was obtained from the child’s medical records.

#### 2.3.2 The parenting stress questionnaires

Maternal parenting stress was assessed using the Chinese version of the Parenting Stress Scale for Parents of Preschool Children, Short Form (CPSS-8), which was developed by Wang et al. (22) to subjectively assess the overall level of parenting stress in parents of preschool children in China. This scale consists of 8 items with a unidimensional factor structure. Higher scores indicate higher levels of parenting stress. The Cronbach’s α coefficient of this scale in the present study was 0.860.

#### 2.3.3 The family hardiness questionnaires

Family hardiness was assessed using the Chinese version of the Family Hardiness Index (FHI) (23). This scale was originally developed by McCubbin and colleagues, and later translated and adapted for the Chinese population by Liu Yang and colleagues to evaluate the family’s capacity for resistance and adjustment in the face of crises. The scale consists of 20 items across three dimensions: commitment, control, and challenge. Items are rated on a 4-point Likert scale from 1 (strongly disagree) to 4 (strongly agree), with 9 items reverse-scored. Higher total scores indicate higher levels of family hardiness. The three dimensions reflect, respectively, the family’s active engagement, belief in taking action under adversity, and view of change as an opportunity for growth. For instance, the “challenge” dimension includes items such as “family members are willing to try new things” and “listen to one another when facing difficulties.” The Cronbach’s *α* coefficient of this scale in the present study was 0.850.

#### 2.3.4 The emotional and behavioral problems questionnaires

Children’s emotional and behavioral problems were assessed using the Strengths and Difficulties Questionnaire (SDQ) developed by Goodman et al. (10). The questionnaire consists of 25 items covering five subscales: emotional symptoms, conduct problems, hyperactivity/inattention, peer relationship problems, and prosocial behavior. The total difficulties score is composed of the emotional symptoms, conduct problems, hyperactivity/inattention, and peer relationship problems subscales. Items are rated on a 3-point scale ranging from 0 (not true) to 2 (certainly true). In this study, the total difficulties score was used to measure emotional and behavioral problems in preschool children, as reported by parents. Higher scores indicate more severe emotional and behavioral problems in children. This questionnaire has demonstrated good reliability and validity in children and adolescents aged 3–16 years (24). The Cronbach’s *α* coefficient of this scale in the present study was 0.771.

### 2.4 Statistical analysis

Statistical analyses were performed using SPSS 27.0, Mplus 8.3, and R 4.5.3. Continuous variables were presented as mean ± standard deviation or median (interquartile range) depending on their distribution, while categorical variables were expressed as percentages. This was an exploratory study. All tests were two-tailed with *α* = 0.05, and no correction for multiple comparisons was applied. Univariate analyses were used for variable screening, and only significant variables were entered into the regression model, with ORs and 95% CIs reported.

*Latent profile analysis* was performed using Mplus 8.3. The mean scores of the four dimensions of emotional and behavioral problems—emotional symptoms, conduct problems, hyperactivity/inattention, and peer relationship problems—were used as the main indicators for model fitting. Starting from a one-class model, we gradually increased the number of latent classes up to five. Model fit was evaluated based on several criteria: the Akaike Information Criterion (AIC), Bayesian Information Criterion (BIC), and adjusted BIC (aBIC), with lower values indicating better fit. The improvement in fit was assessed using the bootstrap likelihood ratio test (BLRT) and the Lo–Mendell–Rubin likelihood ratio test (LMR), where a significant result (*p* < 0.05) indicated that a k-class model fitted better than a *k*-1-class model. In addition, the entropy value was used to evaluate classification accuracy; an entropy value of 0.8 or higher indicated good model fit, with values closer to 1 reflecting more accurate classification. Model selection was based on a comprehensive consideration of AIC, BIC, aBIC, entropy, LMR, BLRT, minimum class proportion (≥5%), and clinical interpretability.

After identifying the subgroups, differences in subgroup characteristics were examined using one-way analysis of variance (ANOVA) for continuous variables, Pearson’s chi-square test or Fisher’s exact test for categorical variables, and the Kruskal-Wallis *H* test for ordinal variables. A *p*-value < 0.05 was considered statistically significant.

*Network analysis (NA)* was used to examine the relationships between children’s emotional and behavioral problems and their influencing factors. Because the data were continuous variables, a Gaussian graphical model was employed to construct the network using the R package “GGM.” In the network, study variables serve as nodes, and the partial correlation coefficients between variables serve as edges. The edge weights represent the strength of direct associations between variables after adjusting for other variables.

The network model was estimated using the EBICglasso algorithm for regularization, which applies the least absolute shrinkage and selection operator (LASSO) for regularization and the extended Bayesian information criterion (EBIC) to guide model selection, with the tuning parameter set to 0.25. Network visualization was performed using the R package “qgraph.” The network layout employed the spring (Fruchterman–Reingold) algorithm, placing strongly associated nodes closer together. In the network graph, thicker lines indicate stronger correlations, blue solid lines represent positive correlations, and red solid lines represent negative correlations.

To identify core nodes in the network, centrality indices were calculated, including node strength, betweenness centrality, and closeness centrality. Among these, node strength is the most central and stable index, representing the sum of all edge weights connected to a node, and is used to determine core variables (25–27). In the node centrality plot, higher values of each centrality index are displayed on the right side.

The R package “bootnet” was used to examine the accuracy and stability of the network model. The accuracy of edge weights was assessed by calculating their 95% confidence intervals. In addition, the correlation stability (CS) coefficient was used to determine the stability of centrality indices, with CS ≥ 0.25 considered acceptable and CS ≥ 0.5 indicating ideal stability. To compare variations in network invariance and global strength invariance across different subgroups, the Network Comparison Test (NCT) was performed using the Network Comparison Test package. The significance level was set at *p* < 0.05.

### 2.5 Ethical considerations

This study was approved by the Ethics Committee of Shandong Provincial Hospital Affiliated to Shandong First Medical University (SWYX: NO.2025-775) and was conducted in accordance with the 1964 Declaration of Helsinki and its later amendments or comparable ethical standards. All participants provided informed consent.

## 3 Results

### 3.1 Participants characteristics

A total of 304 questionnaires were distributed, and 278 were returned. After excluding 5 incomplete questionnaires, 273 valid responses were obtained, yielding an overall completion rate of 89.8%. Among the 273 participating parents, the largest proportion (67.77%) were aged 30–40 years; the largest proportion of children (49.08%) were aged 3–4 years; and the mean emotional and behavioral problem score of the children was 11.89 ± 4.70. Descriptive information on sociodemographic and child medical characteristics is presented in Table 1.

**Table 1:** Differences in demographic and clinical characteristics among subgroups (*n* = 273).

Variables	Total (*n* = 273) *n* (%)/mean ± SD	Class 1 (*n* = 224) *n* (%)/mean ± SD	Class 2 (*n* = 49) *n* (%)/mean ± SD	*χ*^2^/*t*	*p*
Patient characteristic					
Sex				*χ*^2^ = 0.285	0.593
Men	141 (51.65)	114 (80.85)	27 (19.15)		
Women	132 (48.35)	110 (83.33)	22 (16.67)		
Age (year)				*χ*^2^ = 10.938	0.004
3 ~ 4	134 (49.08)	108 (80.60)	26 (19.40)		
4 ~ 5	58 (21.25)	55 (94.83)	3 (5.17)		
5 ~ 6	81 (29.67)	61 (75.31)	20 (24.69)		
CHD classification				*χ*^2^ = 0.242	0.623
Simple congenital heart disease	170 (62.27)	141 (82.94)	29 (17.06)		
Complex congenital heart disease	103 (37.73)	83 (80.58)	20 (19.42)		
Number of cardiac diagnoses				*χ*^2^ = 12.025	0.002
1–2	187 (68.50)	156 (83.42)	31 (16.58)		
3–4	73 (26.74)	62 (84.93)	11 (15.07)		
5 or more	13 (4.76)	6 (46.15)	7 (53.85)		
Timing of diagnosis				*χ*^2^ = 0.122	0.727
Prenatal	78 (28.57)	63 (80.77)	15 (19.23)		
Postnatal	195 (71.43)	161 (82.56)	34 (17.44)		
Number of surgeries				*χ*^2^ = 13.442	<0.001
1 time	255 (93.41)	215 (84.31)	40 (15.69)		
2 times or more	18 (6.59)	9 (50.00)	9 (50.00)		
Number of hospitalizations due to CHD				*χ*^2^ = 5.596	0.018
1 time	222 (81.32)	188 (84.68)	34 (15.32)		
2 times or more	51 (18.68)	36 (70.59)	15 (29.41)		
Mother characteristics					
Age (year)				*χ*^2^ = 3.153	0.207
≤30	53 (19.41)	45 (84.91)	8 (15.09)		
30–40	185 (67.77)	154 (83.24)	31 (16.76)		
≥40	35 (12.82)	25 (71.43)	10 (28.57)		
Education level				*χ*^2^ = 7.229	0.027
Primary school or below	18 (6.59)	11 (61.11)	7 (38.89)		
Junior high/High school	117 (42.86)	94 (80.34)	23 (19.66)		
College/University or above	138 (50.55)	119 (86.23)	19 (13.77)		
Occupation				*χ*^2^ = 5.391	0.145
Unemployed	69 (25.27)	55 (79.71)	14 (20.29)		
Farmer	37 (13.55)	26 (70.27)	11 (29.73)		
Self-employed	29 (10.62)	24 (82.76)	5 (17.24)		
Employee/staff	138 (50.55)	119 (86.23)	19 (13.77)		
Place of residence				*χ*^2^ = 5.673	0.017
Rural	168 (61.54)	128 (76.19)	37 (22.02)		
Urban	108 (39.56)	96 (88.89)	12 (11.11)		
Medical payment method				*χ*^2^ = 7.361	0.007
Urban resident basic medical insurance	120 (43.96)	107 (89.17)	13 (10.83)		
New rural cooperative medical scheme	153 (56.04)	117 (76.47)	36 (23.53)		
Financial support				*χ*^2^ = 0.025	0.873
Yes	48 (17.58)	39 (81.25)	9 (18.75)		
No	225 (82.42)	185 (82.22)	40 (17.78)		
Annual household income				*χ*^2^ = 11.916	0.008
≤50,000	81 (29.67)	59 (72.84)	22 (27.16)		
50,000–100,000	89 (32.60)	72 (80.90)	17 (19.10)		
10,000–150,000	61 (22.34)	53 (86.89)	8 (13.11)		
>150,000	42 (15.38)	40 (95.24)	2 (4.76)		
Number of children				*χ*^2^ = 8.496	0.014
1	64 (23.44)	58 (90.63)	6 (9.38)		
2	164 (60.07)	135 (82.32)	29 (17.68)		
≥3	45 (16.48)	31 (68.89)	14 (31.11)		
Presence of illness in other children in the family				*χ*^2^ = 3.270	0.073
Yes	6 (2.20)	3 (50.00)	3 (50.00)		
No	267 (97.80)	221 (82.77)	46 (17.23)		
Family type				*χ*^2^ = 4.835	0.184
Nuclear family	137 (50.18)	115 (83.94)	22 (16.06)		
Stem family	127 (46.52)	104 (81.89)	23 (18.11)		
Single-parent family	4 (1.47)	3 (75.00)	1 (25.00)		
Blended family	5 (1.83)	2 (40.00)	3 (60.00)		
Child EBPs	11.89 ± 4.70	10.35 ± 3.54	18.92 ± 2.33	*t* = −20.998	<0.001
Parenting stress	18.89 ± 5.14	18.34 ± 4.96	21.39 ± 5.25	*t* = −3.856	<0.001
Family hardiness	58.79 ± 6.98	59.66 ± 6.75	54.82 ± 6.66	*t* = 4.561	<0.001

Complex congenital heart disease includes tetralogy of Fallot, pulmonary atresia, double outlet right ventricle, transposition of the great arteries, hypoplastic left heart syndrome, hypoplastic right heart syndrome, total anomalous pulmonary venous return, complete atrioventricular septal defect, interrupted aortic arch, etc. (48).

### 3.2 Latent profile analysis of emotional and behavioral problems in children with congenital heart disease

In this study, latent class models with 1 to 5 classes were estimated. The fit indices are shown in Table 2. The 2-class model was ultimately selected as the optimal model. First, the BIC of the 2-class model was significantly lower than that of the 1-class model, and both the LMR-LRT and BLRT reached statistical significance, indicating significantly better discrimination of the data than the 1-class model. Second, an entropy value close to 1 is preferred, indicating higher classification accuracy. In our study, the entropy value of the 2-class model was 0.833, which was higher than that of the 3-class model (entropy = 0.713) and the 4-class model (entropy = 0.781). Moreover, although the 3-class and 4-class models showed slight improvements in some information criteria, further increasing the number of classes led to increased model complexity, uneven sample distribution, and reduced clinical interpretability and practicality. For the 5-class model, both the LMR test and BLRT were non-significant. Therefore, the 2-class model achieved the best balance among goodness-of-fit, class separation, classification accuracy, and sample distribution, and was determined as the final model.

**Table 2:** Latent profile model fit indices (*N* = 273).

Profile	AIC	BIC	aBIC	Entropy	LMR (*p*)	BLRT (*p*)	Class probability
1-profile	645.191	674.067	648.701	—	—	—	—
2-profile	498.887	545.81	504.59	0.833	0.0007	<0.0001	0.82051/0.17949
3-profile	452.771	517.742	460.668	0.713	0.0449	<0.0001	0.26007/0.60073/0.13919
4-profile	442.148	525.166	452.238	0.781	0.022	<0.0001	0.27839/0.00366/0.58974/0.12821
5-profile	432.205	533.27	444.489	0.822	0.4319	0.05	0.26740/0.02564/0.00366/0.13553/0.56777

AIC, Akaike information criterion; BIC, Bayesian information criterion; aBIC, adjusted Bayesian information criterion; BLRT, bootstrapped likelihood ratio test; LMR, Lo–Mendell–Rubin likelihood ratio test.

The latent profile analysis results divided the sample into two distinct subgroups. The latent characteristics of the dimensions of children’s emotional and behavioral problems are shown in Figure 1. Class 1 (*n* = 224, 82.1%) had relatively low mean scores on all dimensions, indicating fewer difficulties in children with congenital heart disease. Therefore, we named Class 1 the “well-adjusted subgroup (WA)”. Class 2 (*n* = 49, 17.9%) reported high levels of difficulties in children. Thus, we named Class 2 the “maladjusted subgroup (MA)”.

**Figure 1:**
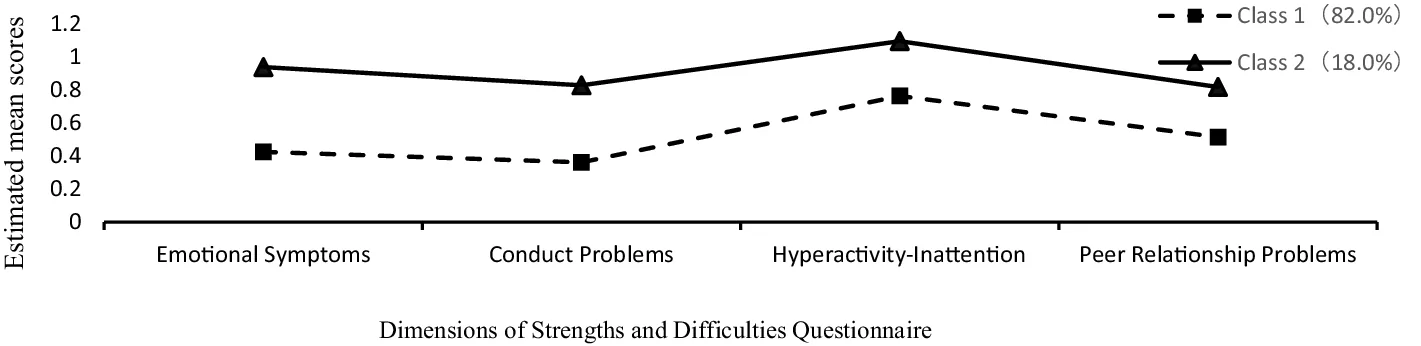
Distribution of estimated mean scores for difficulties questionnaire in the 2-class LPA model.

### 3.3 Heterogeneity of characteristics and emotional and behavioral problems between different subgroups in children with congenital heart disease

The two subgroups showed statistically significant differences in child age, number of cardiac diagnoses, number of hospitalizations due to CHD, number of surgeries due to CHD, maternal education level, place of residence, medical payment method, financial support, annual household income, and number of children in the family (all *p* < 0.05) (Table 1).

There was a significant difference in total difficulties scores of children’s emotional and behavioral problems between the two subgroups (*t* = −20.998, *p* < 0.001), with individuals in the well-adjusted subgroup reporting lower scores than those in the maladjusted subgroup. Similarly, significant differences were found between the two subgroups in maternal parenting stress and family hardiness (both *p* < 0.001). Mothers in the well-adjusted subgroup reported lower parenting stress scores than those in the maladjusted subgroup, whereas individuals in the well-adjusted subgroup reported higher family hardiness scores than those in the maladjusted subgroup.

To evaluate the clinical relevance of the two latent subgroups identified by LPA, we calculated the proportion of children in each subgroup whose SDQ total difficulties score exceeded the established normative cut-off. In the well-adjusted subgroup, only 3 out of 224 children (1.3%) scored above this clinical threshold. In contrast, in the maladjusted subgroup, 40 out of 49 children (81.6%) exceeded the cut-off. This marked difference indicates that the maladjusted subgroup identified by LPA corresponds closely to clinically concerning levels of emotional and behavioral difficulties, whereas the well-adjusted subgroup predominantly represents children without clinically significant problems. These findings strongly support the clinical validity of our LPA classification.

### 3.4 Influencing factors of emotional and behavioral problems in children with congenital heart disease

Variables that were statistically significant in the univariate analysis were entered into a binary logistic regression model, as shown in Table 3. The results showed that maternal parenting stress (OR = 1.086, 95% CI:1.003–1.175, *p* = 0.041) was a risk factor for emotional and behavioral problems in children with congenital heart disease; that is, higher parenting stress was associated with a higher risk of children developing high levels of emotional and behavioral problems. In contrast, family hardiness (OR = 0.907, 95% CI:0.847–0.971, *p* = 0.005) was a protective factor; that is, higher family hardiness was associated with a lower risk of children developing emotional and behavioral problems. The remaining variables, including child age, number of cardiac diagnoses, number of surgeries due to CHD, number of hospitalizations due to CHD, maternal education level, place of residence, medical payment method, annual household income, and number of children in the family, did not show independent effects on the emotional and behavioral problem subgroups (*p* > 0.05).

**Table 3:** Factors associated with emotional and behavioral problem subgroups in children with congenital heart disease.

Variables	*β*	SE	Wald *χ*^2^	OR (95%CI)	*p*
Age (year)					
3 ~ 4	Ref				
4 ~ 5	−0.862	0.679	1.613	0.422 (0.112–1.597)	0.204
5 ~ 6	0.602	0.415	2.103	1.826 (0.809–4.119)	0.147
Number of cardiac diagnoses					
1–2	Ref				
3–4	0.012	0.455	0.001	1.012 (0.415–2.468)	1.012
5 or more	1.190	0.787	2.29	3.288 (0.704–15.362)	0.130
Number of surgeries					
1 time	Ref				
2 times or more	0.758	0.670	1.282	2.135 (0.574–7.938)	0.258
Number of hospitalizations due to CHD					
1 time	Ref				
2 times or more	0.164	0.467	0.123	1.178 (0.472–2.940)	0.725
Education level					
Primary school or below	Ref				
Junior high/High school	−0.344	0.698	0.243	0.709 (0.180–2.786)	0.622
College/University or above	−0.092	0.782	0.014	0.912 (0.197–4.220)	0.906
Place of residence					
Urban	Ref				
Rural	0.081	0.523	0.024	1.084 (0.389–3.023)	0.877
Medical payment method					
Urban resident basic medical insurance	Ref				
New rural cooperative medical scheme	0.491	0.460	1.14	1.634 (0.664–4.024)	0.286
Annual household income					
≤50,000	Ref				
50,000–100,000	−0.44	0.495	0.789	0.644 (0.244–1.700)	0.374
10,000–150,000	−0.791	0.652	1.472	0.453 (0.126–1.627)	0.225
>150,000	−1.644	0.904	3.308	0.193 (0.033–1.136)	0.069
Number of children					
1	Ref				
2	0.21	0.553	0.145	1.234 (0.418–3.644)	0.704
≥3	0.641	0.671	0.912	1.898 (0.510–7.069)	0.339
Parenting stress	0.082	0.040	4.189	1.086 (1.003–1.175)	0.041
Family hardiness	−0.098	0.035	7.930	0.907 (0.847–0.971)	0.005

### 3.5 Networks of emotional and behavioral problems and family psychological environment in different subgroups

To further reveal the interaction mechanisms between the family psychological environment and emotional and behavioral problems in children with congenital heart disease across different subgroups, this study constructed network models within the two subgroups identified by LPA. Each network included parenting stress (PS), the three dimensions of family hardiness (control [FI1], challenge [FI2], and commitment [FI3]), and the four dimensions of children’s emotional and behavioral problems (emotional difficulties [SDQ1], conduct difficulties [SDQ2], hyperactivity/inattention [SDQ3], and peer difficulties [SDQ4]). The network diagrams and centrality indices of the three dimensions of children’s emotional and behavioral problems and their influencing factors are shown in Figures 2, 3.

**Figure 2:**
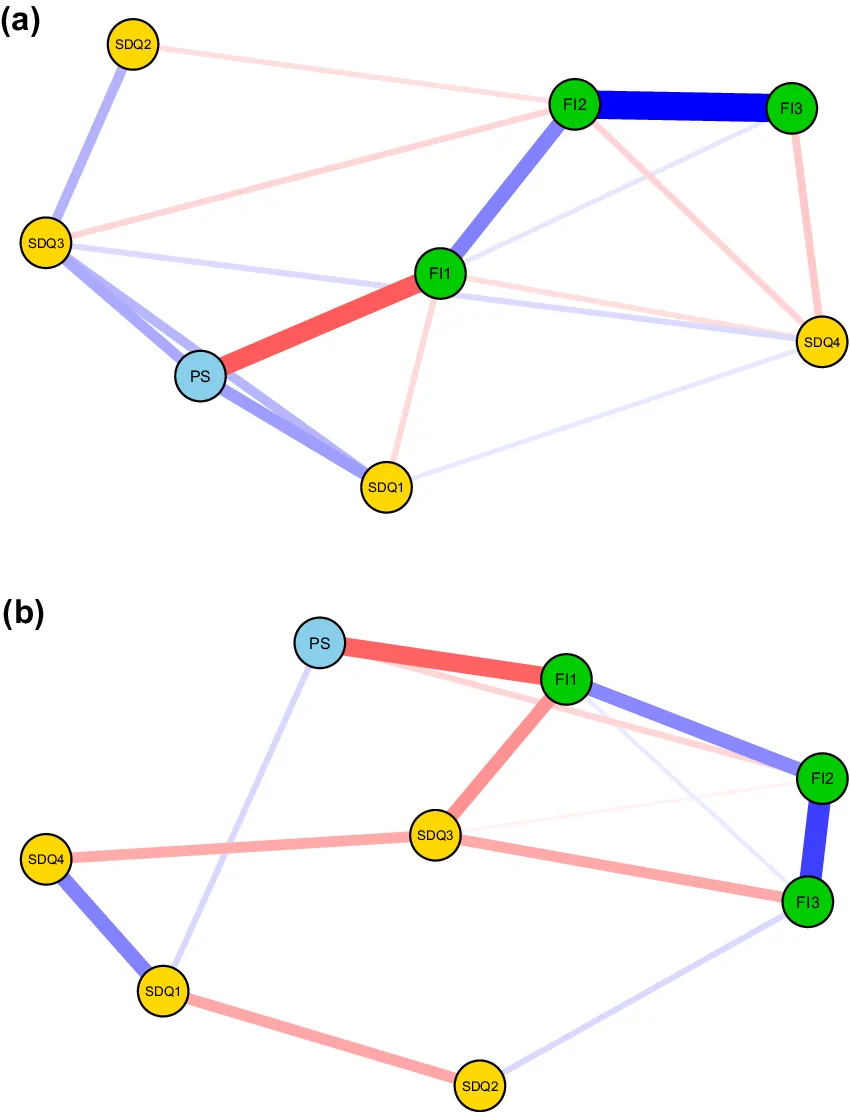
Network diagrams of the two different subgroups of emotional and behavioral problems in children with congenital heart disease. **(a)** Network diagram of the well-adjusted subgroup; **(b)** network diagram of the maladjusted subgroup. Edge thickness indicates the strength of association (thicker edges represent stronger associations), and edge color indicates the nature of the association (blue for positive correlations, red for negative correlations). Network nodes include the following variables: PS, parenting stress; FI1, control dimension; FI2, challenge dimension; FI3, commitment dimension; SDQ1, emotional difficulties; SDQ2, conduct difficulties; SDQ3, hyperactivity/inattention; SDQ4, peer difficulties.

**Figure 3:**
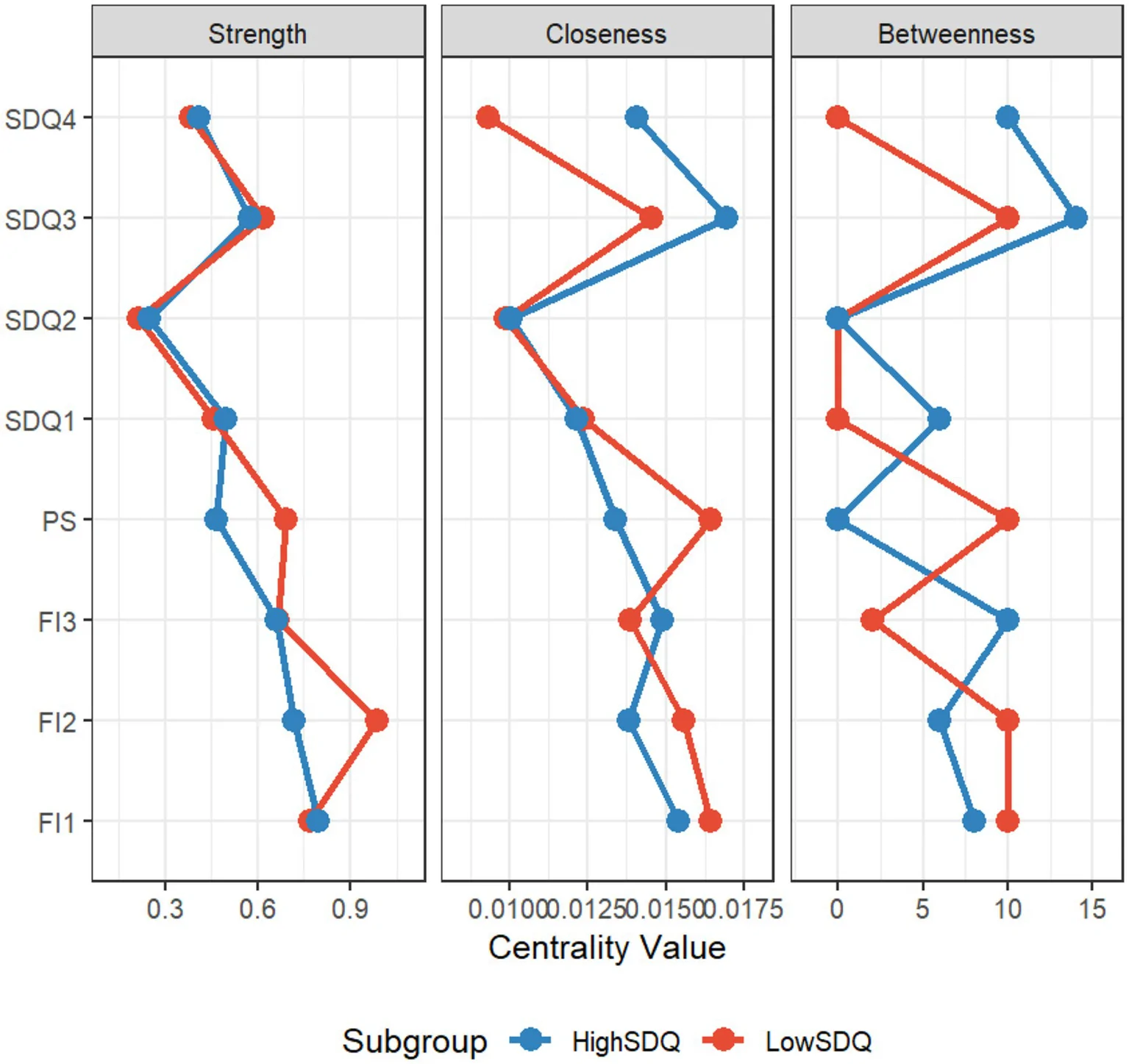
Node centrality plots of the two subgroups of emotional and behavioral problems in children with congenital heart disease. PS, parenting stress; FI1, control dimension; FI2, challenge dimension; FI3, commitment dimension; SDQ1, emotional difficulties; SDQ2, conduct difficulties; SDQ3, hyperactivity/inattention; SDQ4, peer difficulties.

Figure 2a presents the network structure of the well-adjusted subgroup (WA). Among the four different dimensions of emotional and behavioral problems in children with CHD, emotional difficulties (SDQ1) were positively correlated with hyperactivity/inattention (SDQ3) (*r* = 0.142), and conduct difficulties (SDQ2) were positively correlated with hyperactivity/inattention (SDQ3) (*r* = 0.152). Considering the four dimensions of children’s emotional and behavioral problems together with their influencing factors, parenting stress (PS) was positively correlated with emotional difficulties (SDQ1) (*r* = 0.194) and with hyperactivity/inattention (SDQ3) (*r* = 0.168). The control dimension (FI1) was negatively correlated with emotional difficulties (SDQ1) (*r* = −0.076) and with peer difficulties (SDQ4) (*r* = −0.067). The challenge dimension (FI2) was negatively correlated with conduct difficulties (SDQ2) (*r* = −0.065), hyperactivity/inattention (SDQ3) (*r* = −0.081), and peer difficulties (SDQ4) (r − 0.085). The commitment dimension (FI3) was negatively correlated with peer difficulties (SDQ4) (*r* = −0.109). In addition, interactions were also observed among the influencing factors: the challenge dimension (FI2) showed a strong positive correlation with the commitment dimension (FI3) (*r* = 0.506), and the challenge dimension (FI2) also showed a positive correlation with the control dimension (FI1) (*r* = 0.249).

Furthermore, as shown in Figure 3, in the well-adjusted subgroup, hyperactivity/inattention was identified as the core dimension of children’s emotional and behavioral problems (Strength = 0.617, Closeness = 0.015, Betweenness = 10). The challenge dimension (FI2) emerged as a core influencing factor in the well-adjusted subgroup (Strength = 0.986, Closeness = 0.016, Betweenness = 10), revealing the key role of family psychological environment factors in alleviating children’s emotional and behavioral problems.

Figure 2b presents the network structure of the maladjusted subgroup (MA). Among the four different dimensions of emotional and behavioral problems in children with CHD, emotional difficulties were negatively correlated with conduct difficulties (*r* = −0.175) and positively correlated with peer difficulties (*r* = 0.246). Hyperactivity/inattention was negatively correlated with peer difficulties (*r* = −0.166). When examining the influencing factors, maternal parenting stress was positively correlated with children’s emotional difficulties (*r* = 0.076). The hyperactivity/inattention dimension of children’s emotional and behavioral problems was negatively correlated with the control dimension (FI1) (*r* = −0.215), the challenge dimension (FI2) (*r* = −0.025), and the commitment dimension (FI3) (*r* = −0.168). The commitment dimension (FI3) was positively correlated with the conduct difficulties dimension of children (*r* = 0.075). The hyperactivity/inattention dimension (Strength = 0.574, Closeness = 0.017, Betweenness = 14) remained the core dimension among the four dimensions of emotional and behavioral problems in the maladjusted subgroup (Figure 3). Unlike the well-adjusted subgroup, in the maladjusted subgroup, the control dimension (FI1) (Strength = 0.797, Closeness = 0.015, Betweenness = 8) emerged as the core influencing factor.

Network stability testing showed that the bootstrap variations of edge weights were small in each subgroup, indicating good stability of the edge weights within the networks (Supplementary Figures 1, 2). In addition, the correlation stability coefficient for the strength centrality index was 0.594 in the well-adjusted subgroup (Supplementary Figure 3), and 0.205 in the maladjusted subgroup (Supplementary Figure 4), indicating that the centrality indices of the well-adjusted subgroup had moderate stability, whereas those of the maladjusted subgroup had low stability, requiring cautious interpretation of the results. Moreover, no significant difference was found in the network structures between the different emotional and behavioral subgroups (*p* = 0.371), nor was there a significant difference in global strength between the subgroups (*p* = 0.688), suggesting similarity in the overall connectivity patterns of the networks between the two subgroups.

## 4 Discussion

In this study, latent profile analysis (LPA) was used to classify emotional and behavioral problems in children with congenital heart disease into two subgroups: a well-adjusted subgroup and a maladjusted subgroup. Drawing on the family resilience theoretical model, we analyzed the associations between the family psychological environment and children’s emotional and behavioral problems. Furthermore, we constructed a network of children’s emotional and behavioral problems and their influencing factors, which suggested that hyperactivity/inattention was the node with the highest centrality in the network of children’s emotional and behavioral problems, while different dimensions of family hardiness (challenge and control) showed the highest centrality in the well-adjusted subgroup and the maladjusted subgroup, respectively. These findings provide new perspectives for understanding the internal structure of emotional and behavioral problems in children with CHD and their complex associations with the family environment, and offer clues for precise intervention.

### 4.1 Networks of emotional and behavioral problems and family psychological environment in different subgroups

In this study, latent profile analysis classified emotional and behavioral problems in children with congenital heart disease into two heterogeneous subgroups: a well-adjusted subgroup and a maladjusted subgroup. Across the four dimensions of emotional and behavioral problems, children in the maladjusted subgroup scored significantly higher on emotional difficulties, conduct difficulties, hyperactivity/inattention, and peer difficulties than those in the well-adjusted subgroup. This result is consistent with the findings of Dahlawi et al. (8) that complex CHD and multiple surgeries are associated with a higher risk of anxiety, withdrawal, impulsivity, and peer difficulties in children, which aligns with the subgroup distribution characteristics in the present study. Moreover, a systematic review by Latal et al. (6) suggested that children with CHD are at risk for psychological maladaptation across various domains. The development of emotional and behavioral problems in children with CHD shows significant individual differences and does not follow a single developmental trajectory. Therefore, a “one-size-fits-all” approach to assessment and intervention should be abandoned in clinical practice, and stratified, precise management should be implemented based on subgroup characteristics.

More importantly, the present study further revealed differences in the relationships among dimensions of emotional and behavioral problems between the two subgroups through network analysis. In the well-adjusted subgroup, hyperactivity/inattention was positively correlated with peer difficulties, and there were positive associations among emotional difficulties, conduct difficulties, and hyperactivity/inattention, showing a stable co-activation pattern among symptoms. In contrast, in the maladjusted subgroup, hyperactivity/inattention was negatively correlated with conduct difficulties; the original associations among emotional difficulties, conduct difficulties, and hyperactivity/inattention disappeared, and a new pattern emerged in which emotional difficulties were negatively correlated with conduct difficulties. These descriptive differences suggest that the patterns of associations among emotional and behavioral dimensions may differ across subgroups. However, these differences should be interpreted with caution, as the Network Comparison Test did not reveal a statistically significant difference in overall network structure between the two groups. They should therefore be regarded as preliminary findings requiring replication.

Although the Network Comparison Test (NCT) showed no statistically significant difference in overall network structure between the two groups (*P* > 0.05), marked differences were observed in the direction and strength of associations among dimensions within each group. This suggests that the patterns of associations among symptoms may differ in the subgroup with higher severity of emotional and behavioral problems. This is consistent with the view of Cassedy et al. (28) that disease-related chronic stress may be associated with abnormal associations between emotional and behavioral dimensions in children, making the symptom network structure more complex; however, further research is needed to verify this.

Previous studies have suggested that emotional and behavioral problems in children do not exist in isolation but rather interact and influence each other (29). The findings of this study are consistent with those of Lin Nan and colleagues, showing stable positive associations among hyperactivity/inattention, emotional difficulties, and conduct problems: the more prominent the hyperactive behavior, the more likely children are to experience anxiety, withdrawal, and conduct problems (30). Meanwhile, peer relationship problems further affect emotions, leading to increased difficulties in emotional regulation (31). The need for peer belonging is an important part of individual development. When children face peer rejection, they may experience a series of negative events, including internalizing and externalizing problems (32). With regard to internalizing problems, individuals who experience peer rejection often experience greater psychological stress, including more discomfort, feelings of pressure, low self-esteem, loneliness, and helplessness. Consequently, the probability of developing psychological problems such as anxiety and depression increases significantly in this group (50). With regard to externalizing problems, peer rejection may be associated with an increased risk of externalizing behaviors such as aggressive behavior and impulsive behavior in preschool children, and may affect their social participation, peer interaction quality, and early school adaptation (33). The preschool period is a critical stage for emotional and behavioral development, during which the various symptom dimensions form a mutually driven, co-developing pattern of associations (34). In children with congenital heart disease, factors such as disease-related trauma, repeated medical experiences, and family stress may be related to the strength of these associations, and may be associated with a more complex network of associations (28). Therefore, it is essential to closely monitor changes in the four dimensions of children’s emotional and behavioral problems, implement timely interventions, which may help promote their social participation, emotion regulation, and school adaptation, but the effects of such interventions still require further validation.

### 4.2 Core subgroup dimensions of emotional and behavioral problems in children with CHD

Network analysis revealed that hyperactivity/inattention was the core node with the highest strength centrality in both the well-adjusted and maladjusted subgroups, serving as a key hub connecting various emotional and behavioral symptoms. This finding is highly consistent with the rules of child development. The preschool period is a critical stage for the development of inhibitory control, attention, and executive functions in children (12). Children with CHD are more likely to exhibit hyperactive behaviors such as impulsivity, inattention, and restlessness due to the cumulative burden of surgical trauma, hospitalization, or parent–child relationship dynamics (35). Similar patterns have been reported in preterm infants, where levels of attention, social, and emotional problems identified at age 2 years persisted into childhood and even early adulthood (34). In children with congenital heart disease, abnormalities in cerebral perfusion, hypoxia, and altered brain development may exist as early as the fetal period. Combined with perioperative stress, anesthesia exposure, repeated medical stimulation, and early separation, these factors may affect neurodevelopment and executive function, thereby increasing the risk of attention regulation and impulsive–hyperactive behaviors (36). The American Heart Association scientific statement has also clearly indicated that the incidence of attention-deficit/hyperactivity problems is significantly higher in children with CHD than in healthy peers, and attention difficulties are an important associated factor of long-term psychosocial maladjustment (37, 38). Moreover, hyperactivity/inattention is an externalizing behavioral symptom that is intuitive, easily observable, and readily recognized by parents. Compared with internalizing symptoms such as emotional difficulties and peer problems, it is more likely to be prominently reported in scale assessments, which is an important reason why the hyperactivity dimension became a core node in the network. Based on the above findings, hyperactivity/inattention can be considered an important dimension for screening and further evaluation for emotional and behavioral problems in children with CHD. Early behavioral observation, attention assessment, and parent–child interaction guidance may help improve overall adaptation; however, whether interventions targeting this dimension can interrupt symptom transmission or improve overall functioning still requires longitudinal and intervention research for validation.

### 4.3 Factors influencing emotional and behavioral problems in children with CHD

This study found that children in the maladjusted subgroup were older, had a greater number of CHD diagnoses, and underwent more surgeries and hospitalizations; their mothers had lower educational levels and lower annual household incomes, and a higher proportion lived in rural areas or in families with multiple children, suggesting that disease burden and socioeconomic background may be associated with emotional and behavioral adaptation. Existing studies have confirmed that increased number of surgeries, longer hospital stays, and higher disease complexity are associated with an increased risk of psychological stress and emotional-behavioral problems in children with CHD (39, 40). Meanwhile, prolonged medical experiences, repeated hospitalizations, and invasive procedures can become significant stressors for children, further inducing anxiety, withdrawal, impulsivity, and other behavioral abnormalities (41). In addition, lower family socioeconomic status, insufficient maternal education, and limited family financial resources reduce the family’s access to support and intervention resources, thereby increasing the risk of psychological maladaptation in children with CHD. A study by Goldsworthy et al. (42) analyzing 105 2-year-old children with CHD confirmed that lower maternal education was associated with more pronounced externalizing behavior problems and a higher risk of neurodevelopmental delay. This is consistent with the findings of Jilek et al. (43) that lower maternal education is associated with increased behavioral and emotional problems in toddlers with CHD. Binary logistic regression showed that parenting stress was independently associated with emotional-behavioral problems, while family hardiness was independently associated with a lower risk. This phenomenon may be explained by the Resiliency Model of Family Stress, Adjustment, and Adaptation (19). The core tenet of this model is that stressors make the family system vulnerable, but through established functional patterns, appraisal of stressors, integration of internal and external resources, and the interplay between problem-solving and coping, the family ultimately achieves successful adaptation or maladaptation. When maladaptation occurs, new family crises emerge, manifested as emotional and behavioral problems in the child (15). Mothers of children with CHD face long-term disease uncertainty, caregiving burden, and rehabilitation stress, and their parenting stress is significantly higher than that of mothers of healthy children. High parenting stress reduces maternal sensitivity and disrupts parent–child interactions, directly exacerbating children’s emotional and behavioral problems (44). In contrast, family hardiness, as a core capacity of families to cope with adversity, buffers the impact of stress by stabilizing the family atmosphere, promoting positive communication, and enabling effective coping, thereby providing a safe psychological development environment for the child (45). These findings indicate that children’s emotional and behavioral problems are an external manifestation of family system dysfunction, and that parenting stress and family hardiness are associated with children’s emotional and behavioral adjustment. This result is consistent with the findings of Song et al. (46) that family hardiness is negatively correlated with the incidence of psychological and behavioral problems in adolescents. Greater family hardiness promotes positive health outcomes in children (47). Moreover, network analysis further revealed that the challenge dimension of family hardiness was the core influencing factor in the well-adjusted subgroup, suggesting that these families are able to view the illness as a growth opportunity and proactively adjust their coping strategies. In contrast, the control dimension of family hardiness was the core influencing factor in the maladjusted subgroup, suggesting that under adverse conditions, family members in this subgroup hold the belief that they can change life events through their own actions and are able to take action (23). Different dimensions of family hardiness are associated with children’s current emotional and behavioral adjustment. Therefore, these two core dimensions should serve as intervention targets. For the well-adjusted subgroup, their “challenge-oriented” coping experiences can be summarized and disseminated to guide other families in transforming the disease into an opportunity for growth. For the maladjusted subgroup, the positive role of the control dimension should be strengthened by improving disease-related knowledge and setting controllable goals to enhance the family’s sense of mastery. At the same time, parenting stress relief and parent–child interaction guidance should be combined to fully activate the protective role of family hardiness and improve children’s emotional and behavioral adaptation.

### 4.4 Limitations

First, the convenience sampling method used in this study may introduce selection bias, and geographic and demographic limitations may restrict the generalizability of the findings. Future research should adopt stratified sampling to enhance sample representativeness and be conducted across multiple regions to improve external validity. Second, this cross-sectional study could not establish causal relationships between children’s emotional and behavioral problems and these influencing factors. Therefore, longitudinal studies are necessary to better understand these relationships. Finally, due to sample size limitations, the maladjusted subgroup had a relatively small sample size, resulting in low stability coefficients for the relevant analyses in this subgroup, which may to some extent affect the statistical power of subgroup comparisons and core influencing factor analyses. Nevertheless, this study cross-validated the core findings through network analysis and logistic regression, and key influence mechanisms could still be revealed with the current sample size. Future research should expand the sample size, particularly by increasing the proportion of the maladjusted subgroup, to further verify the stability and reliability of the models. In addition, bidirectional or transactional relationships may exist between the family psychological environment and children’s emotional and behavioral problems, and the cross-sectional design cannot determine the direction; medical complexity may also simultaneously affect family functioning and child outcomes. Future longitudinal studies are needed to further verify these relationships.

## 5 Conclusion

This study identified two subgroups of emotional and behavioral adaptation in children with CHD and suggested the key roles of parenting stress and family hardiness in subgroup classification. Notably, network analysis suggested that the challenge dimension and the control dimension were the nodes with the highest centrality in the two groups, respectively, highlighting that differentiated dimensions of family hardiness can serve as candidate targets when formulating emotional and behavioral intervention strategies, although their causal roles and intervention effects still require validation in longitudinal and intervention studies. Furthermore, family hardiness and parenting stress were independently associated with children’s emotional and behavioral adaptation. Therefore, when designing individualized intervention programs for children with CHD who exhibit poor emotional and behavioral adaptation, it is crucial to consider their unique characteristics and family system functioning, with a particular focus on targeted support for family hardiness and scientific management of parenting stress.

## Data Availability

The raw data supporting the conclusions of this article will be made available by the authors, without undue reservation.
