# Supplementary material for: Latent profiles and network analysis of emotional behaviors in preschool children after congenital heart disease surgery
Source: Front Public Health. 2026 Oct 2;14:1914221. doi: 10.3389/fpubh.2026.1914221 (PMC13672223; doi:10.3389/fpubh.2026.1914221)
Supplement: Supplementary file 1 [file Supplementary_file_1.docx]

Supplementary Material

# Supplementary Figure

# **Supplementary Figure 1.** Non-parametric bootstrapping results assessing edge weight stability of the network in the well-adjusted subgroup (bootstrapped confidence intervals of edge weights).The X-axis shows the strength of the edges, and the Y-axis represents the 95% bootstrapped coefficient intervals (CIs) of pair-wise edges.

**Supplementary Figure 2**. Non-parametric bootstrapping results assessing edge weight stability of the network in the maladjusted subgroup.The X-axis shows the strength of the edges, and the Y-axis represents the 95% bootstrapped coefficient intervals (CIs) of pair-wise edges.

**Supplementary Figure 3**. Stability testing of centrality indices (correlation stability coefficient) in the well-adjusted subgroup (CS = 0.594).

**Supplementary Figure 4.** Stability testing of centrality indices (correlation stability coefficient) in the maladjusted subgroup (CS = 0.205; low stability, results should be interpreted with caution).
